# Postponing elective surgery due to COVID-19 did not decrease the oncological surgery rate in Finland

**DOI:** 10.1093/bjs/znab046

**Published:** 2021-02-28

**Authors:** I Kuitunen, V T Ponkilainen, M M Uimonen, J Paloneva, A P Launonen, V M Mattila

**Affiliations:** 1 School of Medicine, University of Eastern Finland, Kuopio, Finland; 2 Emergency Department, Mikkeli Central Hospital, Mikkeli, Finland; 3 Department of Surgery, Central Finland Hospital Nova, Jyväskylä, Finland; 4 Department of Orthopaedics, Tampere University Hospital, Tampere, Finland; 5 Faculty of Medicine and Health Technologies, Tampere University, Tampere, Finland

*Dear Editor*

As nationwide lockdown without curfew was introduced in March 2020, hospitals in Finland postponed elective operations extensively and prepared for a surge in patients with COVID-19. Operating room personnel were redeployed to intensive care, and only emergency and urgent operations were performed, as in many countries^1^. Each hospital decided which operations were secured and which were postponed during the first wave of the pandemic. The common consensus was to secure preparedness for emergency operations and to prioritize oncological procedures. Primary care was reorganized, and resources were allocated to testing and contact tracing. In May, the incidence of COVID-19 began to decrease and restrictions were lifted. Concurrently, the rate of primary care and hospital visits returned to a normal level by the end of the summer.

Health officials warned that cancelled elective visits would cause a backlog that could take years to clear^2^. In Finland, costs of these cancellations have been estimated at €2 billion, along with up to 3 million cancelled secondary-care visits in a population of 5.5 million^3^. Globally, nearly 30 million elective procedures were postponed during the first 12 weeks of the pandemic of which nearly 3 million were cancer operations^4^. A few reports have discussed the potentially detrimental effects of delayed cancer diagnoses. A study^5^ from the UK estimated that a 2-month delay from referral to a specialist visit could lead to a reduction in patient life expectancy of up to 8 months. Furthermore, it has been estimated that the backlog will lead to a 10–20 per cent increase in cancer mortality rate during the next 5 years. The aim of this retrospective cross-sectional study was to describe the influence of the reorganization of healthcare services on the incidence of oncological operations during the COVID-19 pandemic in Finland.

This study was conducted in three Finnish hospitals which provide secondary and tertiary care for 710 000 inhabitants (1/6 of the adult population). The monthly numbers of all elective referrals to surgical units and all operations performed between January 2017 and October 2020 in the participating hospitals were analysed. Surgical operations undertaken in the adult population for ICD-10 C-class diagnoses (malignant tumours) were considered as oncological procedures; all others were included in the non-oncological group. Monthly incidence rates with 95 per cent confidence intervals were calculated per 100 000 persons by the Poisson exact method and comparisons made using incidence rate ratios (IRRs). Statistical analyses were done using R version 4.0.3 (R Foundation for Statistical Computing, Vienna, Austria). Study permissions were obtained from the participating hospitals.

**Fig. 1:**
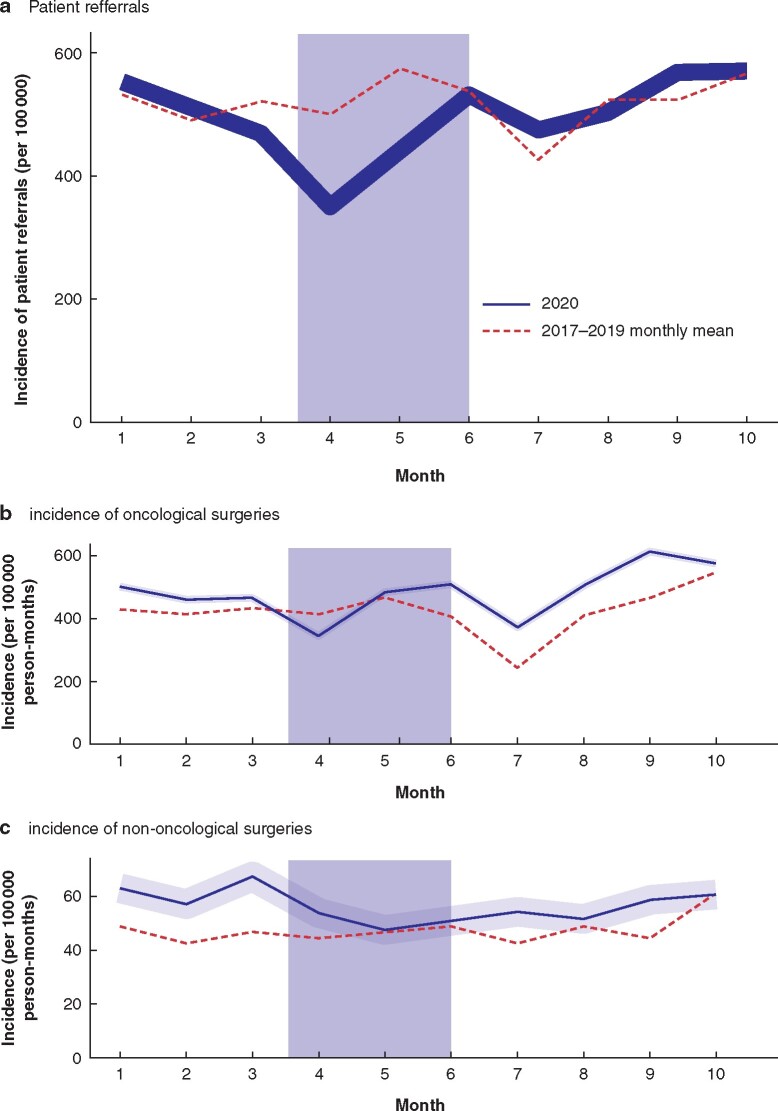
Incidence of patient referrals to surgical units, and oncological and non-oncological operations, in 2020 compared with monthly mean in 2017–2019 **a** Patient referrals to surgical units, **b** incidence of oncological surgeries, and **c** incidence of non-oncological surgeries. Values for 2020 are shown with 95 per cent confidence intervals. The grey shaded area represents the period of nationwide lockdown in Finland.

The incidence of patient referrals to surgical units decreased in March (IRR 0.9, 95 per cent c.i. 0.8 to 1.0) and April (IRR 0.7, 0.6 to 0.7) 2020 compared with the reference years 2017–2019 (*Fig. 1a*). The referral rate returned to the previous level in June (IRR 1.0, 0.9 to 1.0). The incidence of oncological surgery was higher in January (IRR 1.3, 1.2 to 1.4) and February (IRR 1.3, 1.2 to 1.5) 2020 than in 2017–2019. The number of oncological procedures decreased slightly in April and May 2020, but remained at the level of the reference years (IRR 1.0, 0.9 to 1.1) (*Fig. 1b*). The impact of the lockdown was seen prominently in the incidence of non-oncological operations; the incidence was lower in April 2020 (IRR 0.8, 0.8 to 0.9), and higher thereafter than in the reference years (*Fig. 1c*).

The lockdown had a clear impact on the rates of patient referrals to surgical units and non-oncological procedures. The decrease in incidence of oncological operations may have been associated with the decrease in referral rates during the lockdown. Additionally, the participating hospitals prioritized oncological operations over other non-urgent procedures. The COVID-19 mortality rate in Finland is one of the lowest in the world, possibly owing to the low population density and the small mean household size. The low rate of COVID-19 enabled rapid resumption of elective surgery, seen as a rebound in spring, which prevented a backlog of oncological operations. In future, a complete shutdown of healthcare services should be avoided and operating room resources for oncological surgery should be secured during national lockdowns.

*Disclosure.* The authors declare no conflict of interest.
